# Patterns of Daily Physical Activity Across the Spectrum of Visual Field Damage in Glaucoma Patients

**DOI:** 10.1093/geroni/igaa057.2782

**Published:** 2020-12-16

**Authors:** Jennifer Schrack, Jian-Yu E, Aleksandra Mihailovic, Amal Wanigatunga, Sheila West, Laura Gitlin, Tianjing Li, Pradeep Ramulu

**Affiliations:** 1 Johns Hopkins Bloomberg School of Public Health, Baltimore, Maryland, United States; 2 Johns hopkins Bloomberg School of Public Health, Baltimore, Maryland, United States; 3 Johns Hopkins University/Wilmer Eye Institute, Baltimore, Maryland, United States; 4 Johns Hopkins School of Medicine, Baltimore, Maryland, United States; 5 Drexel University, Philadelphia, Pennsylvania, United States; 6 University of Colorado, Arora, Colorado, United States

## Abstract

Visual impairment (VI) has been linked with low physical activity (PA) and higher risk of poor function with aging, yet how VI affects daily patterns of physical activity (PA) is undefined. We assessed the association between visual field (VF) damage and: (i) activity fragmentation and (ii) diurnal patterns of PA in 237 glaucoma patients (mean age=70.6, 51.5% men). PA was measured using the Actical hip-worn accelerometer and VF damage was defined by average VF sensitivity. In adjusted linear models, each 5-dB decrement in IVF sensitivity was associated with 16.3 fewer active minutes/day and 2% higher activity fragmentation (both p<.05). In time-of-day analyses, those with lower IVF sensitivity were less active, but this did not vary by time-of-day. Older adults with worse VF damage demonstrate shorter, more fragmented bouts of PA throughout the day, which may reflect lower physiologic functioning. Future work should examine the temporality of this association.

